# Editorial: Update in pediatric gynecology

**DOI:** 10.3389/fsurg.2024.1391435

**Published:** 2024-03-21

**Authors:** Federica Perelli, Chiara Grimaldi, Massimo Candiani, Lorenzo Masieri, Alberto Mantovani

**Affiliations:** ^1^ Department of Gynecology and Pediatrics, Azienda USL Toscana Centro, Florence, Italy; ^2^ Department of Pediatric Surgery, Meyer Children's Hospital IRCCS, Florence, Italy; ^3^ Obstetrics and Gynecology Department, IRCCS San Raffaele Scientific Institute, Milan, Italy; ^4^ Pediatric Urology Department, Meyer Children's Hospital IRCCS, Florence, Italy

**Keywords:** pediatric gynecology, pediatric surgery, pediatric cancer, Mullerian malformations, fertility sparing surgery, minimally invasive surgery

**Editorial on the Research Topic** Update in pediatric gynecology

## 1 Introduction

Pediatric gynecology represents a crucial issue for the health of girls, adolescents and young women. It involves many branches of medicine and surgery and requires multidisciplinary collaboration between different specialists. Moreover, it represents a complex field both from a diagnostic and therapeutic point of view and in terms of communication, with the young patients and their family.

In the past decades, research has focused on minimally invasive approaches with the aim to save the gonadal function in case of toxic (local and systemic) oncological and non-oncological therapies and prevention of damage to the ovarian reserve following surgery for diseases of gynecological and non-gynecological origin.

Many surgical, malformative, oncological and endocrinological diseases can afflict girls and women from a very early age, starting from the neonatal period.

The pediatric patient requires close collaboration between the pediatrician and the gynecologist, even when the pathology affects non-genital organs but requires surgical or medical therapies for treatment, which can have a negative impact on the endocrinological development and the future fertility of that child.

To preserve the long-term functionality of the genital system, considering the young age of the patients at diagnosis, it is necessary to implement so-called “fertility sparing” diagnostic and therapeutic strategies. The present Research Topic aims to collect innovative research on the current strategies of management of pediatric gynecological patients and the long-term maintenance of their reproductive functions.

## 2 Gynecological implications for disease treatment in children

Pediatric patients and young women affected by diseases that directly or indirectly affect the genital system deserve a multidisciplinary approach, to encompass every aspect of their global health and to ensure the most effective treatment and follow-up.

Congenital malformations of the genitourinary tract frequently require treatment by a team of specialized pediatric urologists and gynecologists who aim to restore normal pelvic anatomy and function, ensuring the best quality of life for the affected patients. Congenital anomalies are usually diagnosed prenatally, but the clinical presentation can often be later in childhood or early puberty, sometimes needing urgent medical or surgical treatment. Thus the importance of (spreading the knowledge of/informing with regard to) these topics, hopefully orienting different-level hospitals to approach the congenital anomaly accurately, regardless of the age or type of presentation, providing the patients with the right approach and recognizing the cases which deserve centralization in a specialized center after the initial management. Therefore, remaining up-to-date seems of particular importance in the field of pediatric gynecological congenital anomalies as research is developing in multiple fields and is predicting enormous advances with innovative surgical techniques that are giving optimal anatomical results (1, 2).

Severe disease affecting the child's vital organs may require medical and surgical treatments that are highly invasive and potentially harmful to overall and genital health. Young girls undergoing organ transplants should benefit from dedicated surgical teams (3). Moreover, it is necessary to include in their follow-up a series of specialists aware of the effects of strong surgical stress and long-term immunosuppressive therapies on their general well-being including future endocrine and reproductive capacity.

Thanks to oncological research, survival following childhood cancer has improved over the past decades and many young cancer patients are living more than 10 years after diagnosis. In this scenario, ovarian preservation achieves maximal importance. Loss of ovarian function after chemotherapy could result in both sterilization and endocrine function deficiency. Fertility preservation is a key component of premature ovarian failure management in children and should be considered for all young patients undergoing potentially gonadotoxic cancer treatments or at high risk for ovarian failure (4).

There are many challenges in implementing pediatric oncology treatment pathways, for example, the need to evaluate accurately the timing of the various types of medical and surgical treatment (surgery, radiotherapy, chemotherapy, preservation of reproductive capacity) and the need to contextualize the epidemiology of female tumors arising at a young age, the diagnostic suspicion and consequently the appropriate diagnostic imaging process and endoscopic procedures (5).

## 3 Pediatric gynecology contribution to improve surgical management of young female patients

This thematic issue evaluated new treatment strategies for young female patients affected by a disease that has to be treated surgically.

Buzinskiene et al. reported the case of a young woman affected by a rare case of ectopic pregnancy located in the interstitial part of the fallopian tube. The authors underlined the need of an accurate, early diagnosis of interstitial pregnancies, based not only on clinical symptoms, but also on additional diagnostic methods by performing a blood test and pelvic ultrasound. Early diagnosis leads to better treatment-related outcomes and makes a conservative treatment possible.

Hu et al. reported a series of 62 children with a median age of 14 months (ranging from 3 to 38 months) with ureteropelvic junction obstruction (UPJO), 32 patients underwent robotic-assisted laparoscopic pyeloplasty (RALP) and the other 30 patients underwent traditional laparoscopic pyeloplasty (LP). All the operations were conducted by the same experienced surgical group from the same hospital. Compared with LP, the data of the RALP group showed a shorter duration of operation, postoperative catheter, and hematuria and recovery but similar clinical efficiency.

Shang et al. reported a series of 20 children with vulvar lichen sclerosus (under 14 years old) enrolled from a single hospital from July 2020 to November 2022 and treated with clitoris exposure + episioplasty + dermabrasion + platelet-rich plasma (PRP) injection + chemexfoliation. The authors registered no local burns or blisters after surgery, but one case of a superficial ulcer, one case of mild vulvar swelling, and one case of minor bleeding from the vulva were observed, with an overall postoperative complication rate of 15% (3/20). The authors concluded that the treatment presented may represent a safe strategy to improve the clinical symptoms effectively and enhance the quality of life of children with vulvar lichen sclerosus.

Perelli et al. presented a series of 311 children and compared the experience of four high-volume European pediatric Centers in terms of surgical outcomes, where oncological female patients at high risk for infertility were submitted for laparoscopic ovarian tissue collection and cryopreservation. The authors showed that the advantage of this technique is that it requires just a few days to be planned and performed, with a minimally invasive approach and, as the retrieval of ovarian tissue is not dependent on the menstrual cycle, no delay in treatment is required. Moreover, their data supported the concept that laparoscopic ovarian tissue collection is a feasible technique which allows the storage of a great number of primordial follicles that are relatively resistant to cryodamage.

## 4 Conclusions

In conclusion, this Research Topic refers to several investigations focusing on relevant surgical aspects of pediatric patients linked to their future genital health.

Advances in pediatric research provide the development of tools for conducting young patient counseling, pre- and post-operative management, and follow-up to tailor the diagnostic, prognostic and therapeutic pathways for each different young female patient through a multidisciplinary approach.

Some other aspects about pediatric gynecology should be addressed and deepened in future Research Topics, such as the fertility sparing surgical therapeutic pathways in young patients with gynecological malignancies, the innovations in surgical and non-surgical treatment of Mullerian malformations, the follow up scheduled for many chronic clinical conditions based on the collaboration between pediatricians, surgeons, urologists, gynecologists and psychologists dedicated to the pediatric patients.
